# The influence of mobile phone self-expansion on psychological richness: a moderated mediation model

**DOI:** 10.3389/fpsyg.2025.1583948

**Published:** 2026-05-18

**Authors:** Guiying Li, Guanghui Yang

**Affiliations:** 1 Henan Polytechnic, Zhengzhou, Henan, China; 2 School of Education, Anyang Normal University, Anyang, Henan, China

**Keywords:** core self-evaluation, mobile phone self-expansion, moderated mediation model, psychological richness, refusal self-efficacy

## Abstract

**Objective:**

Against the background of the widespread use of smartphones and the rising attention to positive psychology, this study examined the impact of mobile phone self-expansion on college students’ psychological richness, as well as the mediating mechanism of core self-evaluation and the boundary condition of refusal self-efficacy.

**Methods:**

Using a cross-sectional quantitative design, 523 college students were surveyed with the Mobile Phone Self-Expansion Scale, Psychological Richness Questionnaire, Core Self-Evaluation Scale, and Refusal Self-Efficacy Scale. Data were analyzed using SPSS 26.0 and Hayes’s PROCESS macro (version 3.5).

**Results:**

(1) Mobile phone self-expansion significantly and positively predicted psychological richness; (2) Core self-evaluation partially mediated the relationship between mobile phone self-expansion and psychological richness; (3) Refusal self-efficacy moderated the first half of the mediation model: the positive predictive effect of mobile phone self-expansion on core self-evaluation was enhanced as refusal self-efficacy increased, and this effect was only significant when refusal self-efficacy was above a certain threshold.

**Conclusion:**

Mobile phone self-expansion affects psychological richness both directly and indirectly through core self-evaluation, and this indirect effect is conditional on refusal self-efficacy. This study enriches the research on the consequences of mobile phone self-expansion and the antecedents of psychological richness.

## 1 Introduction

In recent years, the rise of positive psychology has increased focus on happiness attainment. Psychological richness, a core concept of psychological richness theory (Oishi et al., 2019), is recognized as the third form of happiness after subjective well-being and self-actualization. The theory argues that life’s value lies in the richness and diversity of psychological experiences, not just the pursuit of pleasure or meaning (Oishi et al., 2020; Oishi and Westgate, 2022). It emphasizes that novel, complex, and challenging life experiences enhance psychological richness, which is characterized by novelty, diversity, interest, new perspectives, broadened horizons, variability, uncertainty, complexity, and challenge (Oishi et al., 2019, 2020; Besser and Oishi, 2020). Existing studies confirm that experiences like travel, art appreciation, and challenging learning significantly improve psychological richness (Oishi et al., 2021). However, no research has explored the impact of mobile phone self-expansion on psychological richness. On one hand, mobile phone self-expansion is increasingly prevalent (Hoffner et al., 2016; Wu et al., 2022), and college students—who spend most of their time on campus—may rely on mobile phones to enhance psychological richness. While self-expansion is linked to psychological richness (Wei and Yu, 2024), the specific relationship between mobile phone self-expansion and psychological richness remains unstudied. On the other hand, mobile phone self-expansion is a dual-motivation (Hoffner et al., 2016) with both positive and negative outcomes, yet current research mostly focuses on its negative psychological impacts (disease prevention) rather than positive ones (happiness acquisition), especially regarding psychological richness as a new form of happiness.

This study addresses three core questions: Is there a relationship between mobile phone self-expansion and psychological richness? What is the internal mechanism (does core self-evaluation mediate this relationship)? Under what conditions is this relationship significant (does refusal self-efficacy moderate it)? The core research question is: How does mobile phone self-expansion affect college students’ psychological richness, and what are the mediating and moderating mechanisms? Note that this cross-sectional study only reveals correlations, not causal order—longitudinal studies are needed to verify causality.

## 2 Literature review and hypotheses

### 2.1 The relationship between mobile phone self-expansion and psychological richness

The self-expansion model posits that individuals have an inherent motivation to expand and enhance themselves by absorbing resources, perspectives, and knowledge from external people or objects to promote personal growth (McIntyre et al., 2015; Aron et al., 2013). This theory extends beyond interpersonal relationships to external objects providing resources and experiences. Smartphones, with their ubiquity, interactivity, and rich resources, enable individuals to access information, establish social connections, and engage in diverse activities anytime (Barr et al., 2015; Konok et al., 2016), making them key carriers of external resources aligned with the self-expansion model. Empirical studies confirm that smartphone use facilitates self-expansion, such as improving self-efficacy and broadening social perspectives (Hoffner et al., 2016; Yuan, 2020). Mobile phone self-expansion [consistent with Hoffner et al.’s (2016) “Self-Expansion via Smartphone”] is defined as the motivation and dynamic process where individuals use smartphones to acquire new resources, expand perspectives and experiences, enhance self-identity and abilities, and achieve personal development goals (Hoffner et al., 2016; Wu et al., 2022). It emphasizes active smartphone use for self-growth, covering three core dimensions—new experience acquisition, personal growth, and capability enhancement—and adopts existing theoretical frameworks and measurement tools for consistency. Reasonable mobile phone self-expansion helps individuals access broader perspectives and resources, gaining diverse, novel, and interesting experiences—aligning with psychological richness’s characteristics (novelty, diversity, interest, perspective shift) (Oishi et al., 2019, 2020; Besser and Oishi, 2020). Uncontrolled use may lead to smartphone addiction and negative outcomes (Lizarte Simón et al., 2024; Zhu et al., 2025). Self-expansion correlates positively with psychological richness (Wei and Yu, 2024). In collectivist Chinese culture, college students use mobile phones to maintain relationships and obtain social support (Tian et al., 2025), and mobile phones serve as their key channel to the outside world for diverse information and experiences—providing a situational basis for the relationship. Mobile phone self-expansion also enhances subjective well-being (Yuan, 2020), which correlates positively with psychological richness (Oishi et al., 2019; Wang et al., 2022).

Thus, Hypothesis 1 is proposed: Mobile phone self-expansion positively predicts psychological richness.

### 2.2 The mediating role of core self-evaluation

Core Self-Evaluation is an integrated personality trait that refers to the most basic assessment of one’s abilities and values (Judge and Bono, 2001). It encompasses four aspects: self-esteem, control points, emotional stability, and self-efficacy (Judge and Bono, 2001). Mobile self-expansion not only directly influences psychological richness but may also affect it through mediating factors. Therefore, it is inferred that core self-evaluation could be a mediating variable.

On the one hand, mobile self-expansion may promote core self-evaluation. The self-expansion model posits that individuals have a motivation to expand their potential self-efficacy, with self-expansion aimed at enhancing self-efficacy and competence (Aron et al., 2013). Self-efficacy is a crucial component of core self-evaluation, suggesting that self-expansion might boost it. Empirical studies also find that self-expansion promotes improvements in self-efficacy and self-esteem (Mattingly and Lewandowski, 2012), facilitates personal growth (Mattingly and Lewandowski, 2014), and reduces negative emotional experiences such as burnout and boredom. As a form of self-expansion, mobile self-expansion may also enhance core self-evaluation. Research confirms that posting content on social media can shape individuals perceptions of themselves. Accessing information via mobile devices makes people feel their cognitive abilities are expanded (Barr et al., 2015), provides a sense of security (Konok et al., 2016), and the experiences gained through mobile devices can promote the integration of self-concept (Hoffner et al., 2016). According to the theory of mobile self-expansion (Hoffner et al., 2016; Niu, 2017), individuals experience a rich and diverse life through mobile use, with their social perspectives broadening, life experiences expanding, and knowledge and skills continuously improving, value goals are constantly achieved, basic psychological needs are met, self-efficacy is higher, and self-evaluation is more positive. At present, although there is no research on the relationship between mobile phone self-expansion and core self-evaluation, it is inferred from the concepts of both and relevant empirical studies that mobile phone self-expansion can positively predict core self-evaluation.

On the other hand, core self-evaluation also promotes psychological richness. According to self-cognition theory (Judge et al., 1997), individuals with high core self-evaluation have higher confidence and self-efficacy, are more willing to try rich and interesting experiences, activities, and challenges to further maintain or enhance positive self-concepts; according to cognitive coping theory, individuals with high core self-evaluation dare to proactively expand their lives and pursue goals, have greater ability and confidence to face difficulties and challenges, which may promote the enhancement of psychological richness. Empirical research has found that individual self-esteem and innovative self-efficacy (Wang et al., 2022) are significantly positively correlated with psychological richness, while self-esteem and self-efficacy are important components of core self-evaluation (Judge and Bono, 2001); individuals with high core self-evaluation have higher life satisfaction and subjective well-being (Sun, 2021), are more willing to take risks in decision-making (Hönl et al., 2020), are more likely to seek help proactively and respond positively when in distress (Sun and Mao, 2013), perceive higher social support (McNall and Michel, 2016), and can predict future-oriented optimism, planning, and execution (Tan, 2022), all of which are important conditions for enhancing psychological richness.

Accordingly, hypothesis 2 is proposed that core self-evaluation may play a mediating role between mobile phone self-expansion and psychological richness.

### 2.3 The moderating effect of refusal self-efficacy

Although mobile self-expansion may promote the enhancement of core self-evaluation, not all motives for mobile self-expansion can boost positive effects such as increased core self-evaluation. Research has found that individuals who use their phones negatively, i.e., those with unhealthy mobile self-expansion, experience more negative issues due to their addiction to their phones, such as privacy breaches, phone addiction, loneliness, depression and anxiety, and cognitive impairment (Chen et al., 2016; Yang et al., 2019). For those addicted to their phones, the higher the motive for mobile self-expansion, the poorer the attention control, and the more severe the unconscious procrastination (Liu et al., 2024). Issues like phone addiction, loneliness and depression, cognitive impairment, and procrastination are significantly negatively correlated with core self-evaluation (Chen et al., 2024; Xiang et al., 2021). Therefore, unhealthy mobile self-expansion can lead to phone addiction, resulting in a decline in core self-evaluation. The risk buffering model posits that protective factors can buffer the relationship between risk factors and problem behaviors. Mobile self-expansion is just one motive; whether it promotes individual self-identity and growth depends on whether individuals can resist the temptation of their phones and curb their desires to prevent phone addiction. Refusal self-efficacy may play a moderating role. Refusal self-efficacy refers to an individual’s confidence or ability to resist a certain problem behavior. Refusal self-efficacy is considered the most important individual cognitive factor in preventing problem behaviors (such as smoking, drinking, drug abuse, and smartphone addiction) and predicting healthy behavior (Oei and Jardim, 2007). It is hypothesized that the interaction between refusal self-efficacy (protective factors) and self-expansion of smartphones (risk tendency factors) may reduce the likelihood of adverse outcomes like smartphone addiction (problem behaviors). Empirical studies have found that high levels of refusal self-efficacy can help college students maintain positive emotions; individuals with high refusal self-efficacy can resist negative online temptations, use smartphones reasonably and effectively, and reduce the likelihood of smartphone addiction (Hu, 2023; Geng, 2019; Xu et al., 2012), promoting positive self-expansion of smartphones and thereby reducing negative self-evaluations. Based on this, Research Hypothesis 3 proposes: refusal self-efficacy moderates the relationship between self-expansion of smartphones and core self-evaluation, meaning that individuals with higher refusal self-efficacy have stronger predictive power or more predictive power for self-expansion of smartphones on core self-evaluation.

**FIGURE 1:**
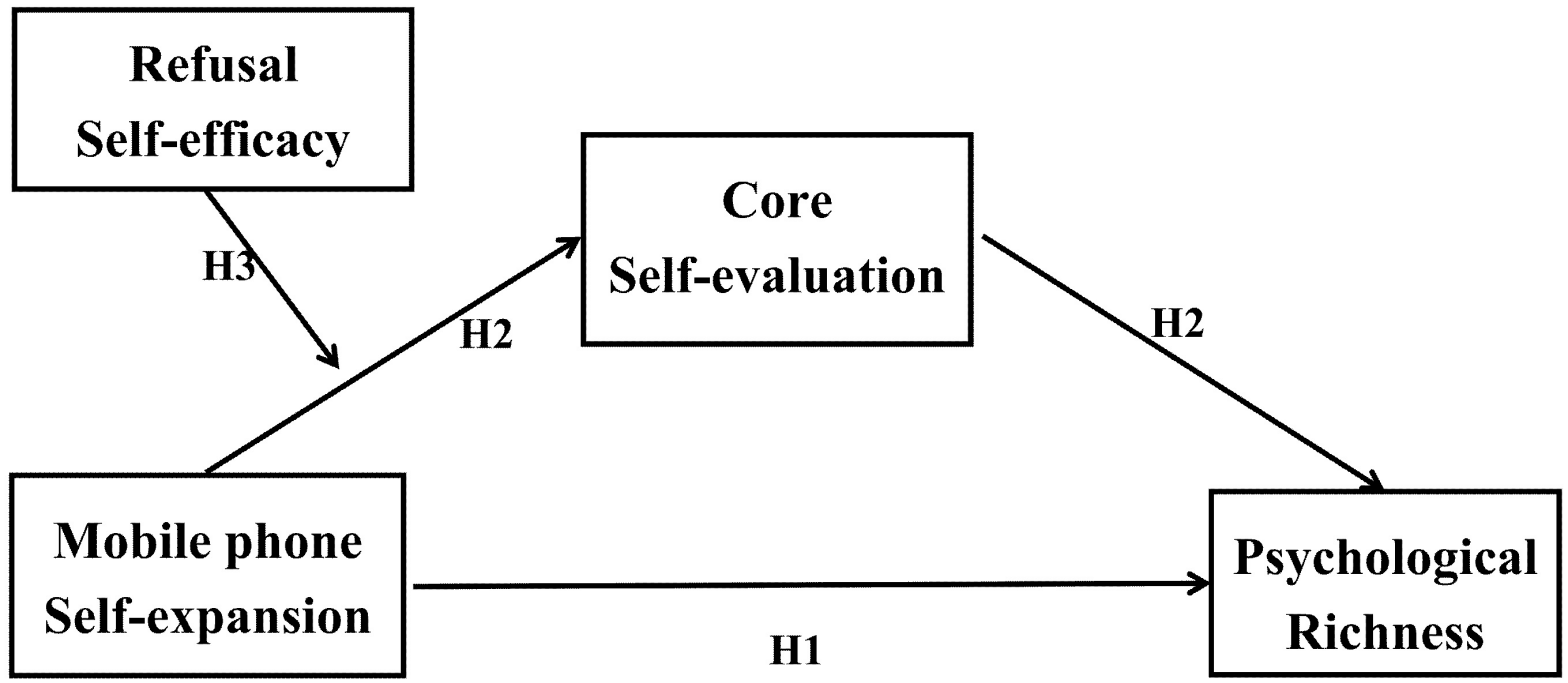
Hypothetical moderated mediation model: the mediating role of core self-evaluation and the moderating role of refusal self-efficacy between mobile phone self-expansion and psychological richness.

To sum up, this study takes college students as the research object to investigate the influence of college students mobile phone self-expansion on psychological richness, as well as the mediating role of core self-evaluation between mobile phone self-expansion and psychological richness and the moderating role of refusal self-efficacy in the first half of the above mediation (as shown in Figure 1).

## 3 Materials and methods

### 3.1 Subjects and the investigation process

In December 2024, students from two universities in Henan were conveniently selected as research subjects. To ensure the effectiveness and authenticity of the data collection, full-time mental health education teachers at their respective schools provided unified guidance in mental health education classes. They explained to the participants the confidentiality of the questionnaire, how to fill it out, and important points to note. After the participants understood, they were individually assigned through the classroom learning software “Yizhijiao” to complete the collective test on the Questionnaire Star platform, with supervision throughout the session. The entire testing process took about 5 min. Among the responses, those with significantly shorter answering times, identical answers, or obvious patterns were excluded, resulting in 523 valid questionnaires. Of these, 247 were male (47.2%) and 276 were female (52.8%). In terms of average daily mobile phone usage: 2 individuals (0.4%) used their phones for less than 30 min, 46 individuals (8.8%) used them for 30 to 2 h, 142 individuals (27.2%) used them for 2 to 4 h, 188 individuals (35.9%) used them for 4 to 6 h, and 145 individuals (27.7%) used them for more than 6 h. This usage distribution is similar to the results of national college student mobile phone use surveys (e.g., Wang et al., 2022), indicating that the sample has a certain degree of representativeness. In addition, the study adopted a convenient sampling method covering two universities in Henan Province, involving multiple majors such as liberal arts, science, and engineering, which further improves the representativeness of the sample to a certain extent.

### 3.2 Measurement tools

#### 3.2.1 Self-expansion scale for mobile phones

The localized version of the mobile self-expansion scale was developed by Yuan (2020) and Niu (2017), referencing Mobile Self-Expansion Scale (Hoffner et al., 2016) and Intimate Relationship Self-Expansion Scale (Aron et al., 2013). The questionnaire consists of 16 items, covering three dimensions: new experiences gained, sense of growth, and enhanced capabilities, scored on a 7-point scale from 1 (strongly disagree) to 7 (strongly agree). Higher individual scores indicate a higher degree of mobile self-expansion. In this study, the Cronbachs α coefficients for the three subscales—new experiences gained, sense of growth, and enhanced capabilities—were 0.922,0.891, and 0.882, respectively, while the overall scales Cronbach’s α coefficient was 0.959.

#### 3.2.2 Psychological richness questionnaire

The short version of the Chinese Psychological Richness Scale, compiled by Oishi et al. (2019) and revised by Wang et al. (2022), consists of 12 items that fully cover the core facets of psychological richness (novelty, diversity, interest, new perspectives, broad horizons, variability, uncertainty, complexity, and challenge of life experiences). Oishi et al. (2019) verified through factor analysis and content validity testing that these 12 items can effectively represent all core dimensions of psychological richness. The scale is scored on a 7-point scale from 1 (strongly disagree) to 7 (strongly agree), forming a single-dimensional self-rating scale, with higher individual scores indicating higher psychological richness. In this study, the Cronbach’s α coefficient for the scale was 0.951.

In terms of the difference from the Mobile Phone Self-Expansion Scale: (1) Construct connotation: Psychological richness focuses on the subjective experience and perception of life experiences, reflecting the diversity and novelty of individuals’ psychological world; mobile phone self-expansion focuses on the motivation and process of using mobile phones to achieve self-growth, reflecting the behavioral tendency of individuals to use mobile phone resources. (2) Measurement focus: The Psychological Richness Scale measures the overall level of individuals’ psychological experience, while the Mobile Phone Self-Expansion Scale measures three specific dimensions related to mobile phone use (acquisition of new experiences, sense of growth, enhanced capabilities). To exclude the risk of collinearity, we conducted a variance inflation factor (VIF) test on the two variables. The results showed that the VIF value of mobile phone self-expansion was 1.23, and the VIF value of psychological richness was 1.31, both less than the critical value of 10, indicating that there is no serious collinearity between the two variables.

#### 3.2.3 Core self-evaluation scale

The scale was developed by Judge et al. (2006) and revised by Du et al. (2012) based on China’s specific conditions. The scale consists of 10 items, scored on a 5-point scale from 1 (strongly disagree) to 5 (strongly agree), making it a single-dimensional self-rating scale. Higher scores indicate higher core self-evaluations. In this study, the Cronbach’s α coefficient for the scale was 0.881.

#### 3.2.4 Refusal self-efficacy scale

The Chinese version of the scale was revised by Xu et al. (2012) based on the scale developed by Wills et al. (2003). The scale consists of 5 items, scored on a 5-point scale from 1 (strongly disagree) to 5 (strongly agree), forming a single-dimensional self-rating scale. A higher total score indicates a stronger sense of refusal self-efficacy among participants. The internal consistency of this scale was assessed in this study using the Cronbach’s α coefficient, which is 0.784.

### 3.3 Data processing

Statistical analysis was conducted using SPSS 26.0, and moderated mediation effect was tested using SPSS macro program PROCESS V3.5. Since the scales used in this study adopted different scoring systems (7-point and 5-point scales), z-score transformation was performed on all scale scores before data analysis to standardize the data.

## 4 Results

### 4.1 Common method bias test

The Harman single-factor method was used to test the common method bias (Xiong et al., 2012). The results showed that there were seven factors with characteristic roots greater than 1, and the first factor explained 36.77% of the variance, which was less than the critical value of 40%, indicating that there was no serious common method bias in this study.

### 4.2 Descriptive statistics and correlation analysis

A correlation analysis was conducted on mobile phone self-expansion, psychological richness, core self-evaluation, and refusal self-efficacy (see Table 1). The results show that mobile phone self-expansion is significantly positively correlated with both core self-evaluation and psychological richness, as well as refusal self-efficacy. Gender has only a significant positive correlation with refusal self-efficacy. Average daily screen time is significantly negatively correlated with mobile phone self-expansion, core self-evaluation, psychological richness, and refusal self-efficacy. Therefore, in subsequent moderated mediation tests, gender and average daily screen time were used as control variables.

**TABLE 1:** The mean (*M*), standard deviation (*SD*), and correlations of the variables (*n* = 523).

Variable	*M*	*SD*	1	2	3	4	5	6
1. Gender	1.530	0.500	1					
2. Daily phone usage	3.820	0.951	0.001	1				
3. Mobile self-expansion	5.174	0.900	−0.035	−0.149**	1			
4. Core self-evaluation	2.870	0.622	−0.015	−0.179***	0.443***	1		
5. Psychological richness	4.924	1.225	−0.075	−0.184***	0.500***	0.549***	1	
6. Refusal self-efficacy	3.910	0.816	0.153**	−0.105*	0.132**	0.320***	0.223***	1

Male = 0, female = 1;

* *P* < 0.05,

** *P* < 0.01,

*** *P* < 0.001, the same below.

### 4.3 Test of moderated mediation model

First, using the Model 4 from Hayes (2013) in the SPSS macro program PROCESS 3.5, we tested the mediating role of mobile phone self-expansion on psychological richness through core self-evaluation. After controlling for gender and average daily mobile phone usage time, mobile phone self-expansion significantly positively predicted core self-evaluation (β = 0.426, *t* = 10.772, *P* < 0.001), mobile phone self-expansion significantly positively predicted psychological richness (β = 0.311, *t* = 8.108, *P* < 0.001), and core self-evaluation significantly positively predicted psychological richness (β = 0.398, *t* = 10.324, *P* < 0.001). The mediating effect of core self-evaluation on the relationship between mobile phone self-expansion and psychological richness is significant, with a 95% confidence interval of [0.120,0.223], and the mediation effect (0.169) accounts for 35.135% of the total effect (0.481) (see Table 2).

**TABLE 2:** Total effect, direct effect and indirect effect among the variables (standardized coefficient).

Effect	Effect size	Boot SE	95%CI	Relative effect size
Total effect	0.481	0.038	[0.406, 0.556]	
Direct effect	0.311	0.038	[0.236, 0.387]	64.865%
Indirect effect	0.169	0.026	[0.120, 0.223]	35.135%

Secondly, all variables were standardized and adjusted for gender and average daily mobile phone usage time before conducting moderated mediation tests using Model 7 from the SPSS macro program PROCESS 3.5. The results (see Table 3) show that the self-expansion of mobile phones significantly predicts core self-evaluation (β = 0.372, *t* = 9.790, *P* < 0.001), and it also significantly predicts psychological richness (β = 0.311, *t* = 8.108, *P* < 0.001). The prediction effect of core self-evaluation on psychological richness is also significant (β = 0.398, *t* = 10.324, *P* < 0.001), and the moderating effect of core self-evaluation remains valid. The prediction effect of refusal self-efficacy on core self-evaluation is significant (β = 0.273, *t* = 7.205, *P* < 0.001), and the interaction term between self-expansion of mobile phones and refusal self-efficacy also significantly predicts core self-evaluation (β = 0.124, *t* = 3.761, *P* < 0.001), with a 95% confidence interval of [0.059,0.189], indicating that refusal self-efficacy moderates the impact of self-expansion of mobile phones on core self-evaluation, and the moderating effect of refusal self-efficacy is significant.

**TABLE 3:** Test of moderated mediation effect of mobile phone self-expansion on psychological richness.

Variable	Dependent variable: core self-evaluation			Dependent variable: psychological richness		
	*β*	*t*	95%*CI*	*β*	*t*	95%*CI*
Sex	−0.077	−1.025	[−0.224, 0.070]	−0.116	−1.693	[−0.251, 0.019]
Daily phone usage	−0.102	−2.575*	[−0.179, −0.024]	−0.070	−1.895	[−0.142, 0.003]
Mobile self-expansion (X)	0.372	9.790***	[0.298, 0.447]	0.311	8.108***	[0.236, 0.387]
Core self-evaluation (M)				0.398	10.324***	[0.322, 0.474]
Refusal self-efficacy (W)	0.273	7.205***	[0.199, 0.348]			
X × W	0.124	3.761***	[0.059, 0.189]			
*R* ^2^	0.295			0.391		
*F*	43.295***			82.983***		

*P < 0.05,

**P < 0.01,

***P < 0.001.

**FIGURE 2:**
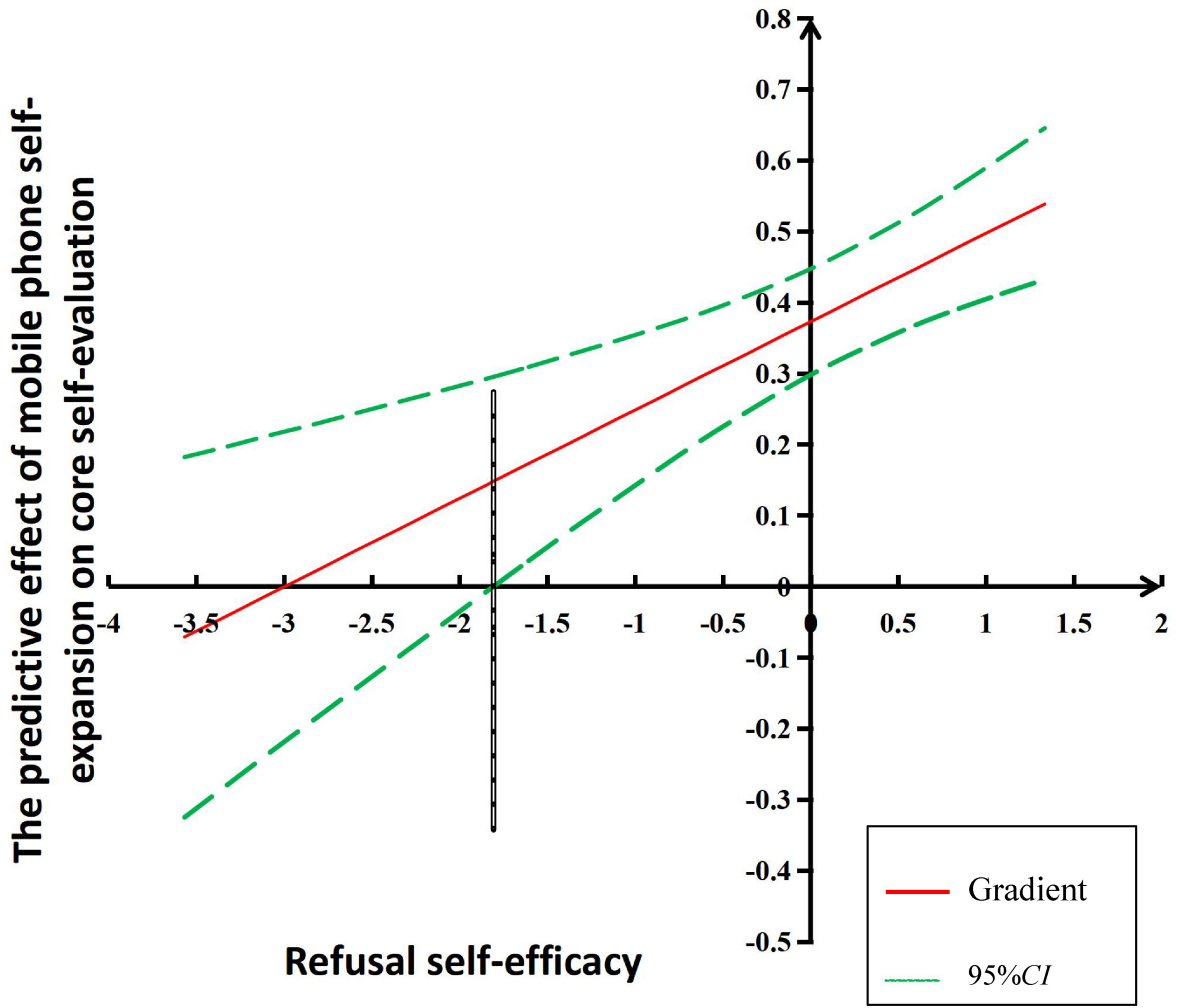
Simple slope analysis of the moderating effect of refusal self-efficacy on the relationship between mobile phone self-expansion and core self-evaluation.

Since the moderating variable (refusal self-efficacy) of this study is a continuous variable, the Johnson-Neyman technique can be adopted to better understand the simple slope change process (Hayes, 2013) The results show that when the refusal self-efficacy is below −1.806 (standard value), the predictive effect of mobile self-expansion on core self-evaluation is not significant; when the refusal self-efficacy is above −1.806, the predictive effect becomes significant. Moreover, the predictive effect of mobile self-expansion on core self-evaluation increases with the rise in college students refusal self-efficacy levels. Specifically, for every one-unit increase in refusal self-efficacy, the positive effect of mobile self-expansion on core self-evaluation increases by 0.13 (see Figure 2).

## 5 Discussion

This study proposes a moderated mediation model to investigate how college students’ mobile phone self-expansion affects psychological richness and under what conditions this effect exists or is even stronger. The results show that mobile phone self-expansion is significantly positively correlated with psychological richness, and core self-evaluation plays a partial mediating role in the impact of mobile phone self-expansion on psychological richness; the moderated mediation analysis indicates that only when refusal self-efficacy is high does the positive predictive effect of mobile phone self-expansion on core self-evaluation become significant, allowing mobile phone self-expansion to influence psychological richness through its impact on core self-evaluation.

### 5.1 The relationship between mobile phone self-expansion and psychological richness

This study found that mobile phone self-expansion is positively correlated with psychological richness; that is, the stronger the motivation for mobile phone self-expansion, the higher the psychological richness, supporting Hypothesis 1 and aligning with related research findings (self-expansion and psychological richness are correlated) (Wei and Yu, 2024). It also validates the related concepts and theories of self-expansion, mobile phone self-expansion, and psychological richness. The self-expansion model posits that individuals have the motivation to incorporate novel and interesting resources and experiences into their self to expand their capabilities and enhance themselves (McIntyre et al., 2015; Aron et al., 2013). According to the concept of mobile phone self-expansion, individuals tend to enhance themselves by obtaining novel, interesting, stimulating, complex, and challenging perspectives, resources, and experiences through their phones (Hoffner et al., 2016; Wu et al., 2022). These novel and interesting gains can increase psychological richness (Oishi and Westgate, 2022). Currently, there is limited research on mobile phone self-expansion and psychological richness in China. Previous studies have shown that self-expansion is related to psychological richness (Wei and Yu, 2024). This study empirically demonstrates that mobile phone self-expansion is also related to psychological richness, expanding the understanding of self-expansion, mobile phone self-expansion, and psychological richness related research content.

### 5.2 Partial mediating role of core self-evaluation

This study found that core self-evaluation (cognitive factors) partially mediates the relationship between mobile phone self-expansion (behavioral motivation) and psychological richness (happiness experience), revealing the internal mechanism by which mobile phone self-expansion influences psychological richness. Specifically, mobile phone self-expansion can directly affect psychological richness or enhance it through improved core self-evaluation, supporting Hypothesis 2. The mediation effect test also validated the self-expansion model, confirming that self-expansion indeed promotes personal growth and enhances self-efficacy (Aron et al., 2013). It further confirmed the mobile phone self-expansion model and the self-cognition theory of core self-evaluation, showing that individuals gain new knowledge, skills, and experiences through mobile phone self-expansion, which increases their self-efficacy and self-esteem (Mattingly and Lewandowski, 2012; Mattingly et al., 2014; Hoffner et al., 2016), reduces negative emotions such as boredom and fatigue, thereby enhancing core self-evaluation (Chen, 2023). Additionally, it validates the cognitive coping theory, indicating that individuals with high core self-evaluation are more willing, capable, and proactive in facing various complex and interesting challenges (Hönl et al., 2020; Sun and Mao, 2013; McNall and Michel, 2016; Tan, 2022), thus enhancing psychological richness.

### 5.3 Moderating effect of refusal self-efficacy

This study also demonstrates that refusal self-efficacy moderates the relationship between mobile phone self-expansion and core self-evaluation. Refusal self-efficacy moderates the first half of the mediating process through which mobile phone self-expansion influences psychological richness via core self-evaluation, supporting Hypothesis 3. This is consistent with previous research findings, indicating that refusal self-efficacy is a significant cognitive factor in preventing mobile phone addiction (Hu, 2023; Geng, 2019), thus validating the social cognitive theory of efficacy. Mobile phone self-expansion is merely a behavioral motivation; its positive or negative impact on individuals depends on their refusal self-efficacy. Only at a certain level of refusal self-efficacy does mobile phone self-expansion have a positive predictive effect on core self-evaluation, thereby enhancing psychological richness. Using the J-N method to examine simple efficiency change trajectories, this study found that when the level of refusal self-efficacy exceeds −1.806 (standard value), mobile phone self-expansion has a positive predictive effect on core self-evaluation. Social cognitive theory posits that an individuals sense of efficacy plays a crucial role in the acquisition, maintenance, and reduction of problem behaviors. Individuals with poor refusal self-efficacy find it difficult to control their time and desire for mobile phone self-expansion, making them more prone to mobile phone addiction. The anxiety, depression, loneliness, and procrastination caused by mobile phone addiction can lower the level of core self-evaluation (Chen et al., 2016; Yang et al., 2019; Liu et al., 2024), which reduces or even eliminates the self-expansion function of mobile phones.

### 5.4 Implications

Previous studies have paid more attention to the adverse effects of the self-expansion of mobile phones, focusing on the prevention of the occurrence of undesirable problems. However, mobile phone self-expansion has both advantages and disadvantages. This study explores the impact and internal mechanisms of mobile phone self-expansion on the third form of happiness (psychological richness) from a positive psychology perspective, based on the mobile phone self-expansion model, self-cognition theory, and psychological richness theory. It also examines the mediating role of core self-evaluation and the moderating effect of refusal self-efficacy. This research expands and deepens existing studies on mobile phone self-expansion and psychological richness, supporting and validating relevant theories. The findings not only provide new insights into enhancing the psychological richness of college students but also offer references for understanding the influence of mobile phone self-expansion tendencies on individual cognition and behavior.

From a practical perspective, this study offers important insights for educators and relevant institutions: First, mobile phone self-expansion can enhance college students’ psychological richness, so schools can incorporate digital literacy education into daily teaching, guiding students to use mobile phones to obtain positive experiences such as knowledge learning and interpersonal communication, and giving full play to the positive role of mobile phone self-expansion. Second, colleges and universities can carry out student well-being improvement programs, including training on refusal self-efficacy and core self-evaluation enhancement, to help students improve their ability to resist mobile phone addiction, enhance their self-recognition and self-efficacy, and thus promote the improvement of psychological richness. Third, families should cooperate with schools to guide adolescents to use mobile phones reasonably, balance the time spent on mobile phone use and offline activities, and create a healthy living environment for the improvement of psychological richness.

### 5.5 Limitations and future study

This study still has some shortcomings, and corresponding future research directions are proposed as follows: First, the cross-sectional design of this study cannot establish the causal order between variables (limitation). Therefore, future research could adopt a longitudinal design to track the dynamic changes of mobile phone self-expansion, core self-evaluation, and psychological richness over time, so as to clarify the causal relationship between variables (corresponding suggestion). Second, the data were obtained through self-report methods, which may be affected by social desirability and lead to inaccurate results (limitation). Future studies could combine multiple measurement methods such as peer evaluation and behavioral observation to collect data, improving the reliability and validity of the data (corresponding suggestion). Third, this study did not distinguish between different types of mobile phone self-expansion (such as entertainment-oriented, learning-oriented, and social-oriented) (limitation). Future research could explore the impact mechanisms of different types of mobile phone self-expansion on psychological richness, so as to provide more targeted practical suggestions (corresponding suggestion). Fourth, this study only focused on mobile phone self-expansion and did not consider the interaction between daily life self-expansion (such as offline social activities, practical learning) and mobile phone self-expansion (limitation). Future studies could integrate these two types of self-expansion into the research model to explore their interactive effects on college students’ psychological richness, which is more in line with the actual situation of individuals’ self-development.

## 6 Conclusion

This study explored the relationship between mobile phone self-expansion and psychological richness among college students, as well as the mediating role of core self-evaluation and the moderating role of refusal self-efficacy, and obtained the following main conclusions: (a) Mobile phone self-expansion significantly and positively predicts the level of psychological richness, which verifies that reasonable mobile phone self-expansion can bring positive psychological experiences such as novelty and diversity to individuals, enriching their psychological world. (b) Core self-evaluation plays a partial mediating role between mobile phone self-expansion and psychological richness, indicating that mobile phone self-expansion can not only directly affect psychological richness but also indirectly promote it by enhancing individuals’ core self-evaluation (including self-esteem, self-efficacy, etc.). (c) Refusal self-efficacy moderates the first half of the mediation model: at low levels of refusal self-efficacy, mobile phone self-expansion has no significant predictive effect on core self-evaluation; only at high levels of refusal self-efficacy can mobile phone self-expansion significantly improve core self-evaluation.

## Data Availability

The raw data supporting the conclusions of this article will be made available by the authors, without undue reservation.
